# Vaccine-induced thrombotic thrombocytopenia (VITT): first report from India

**DOI:** 10.1186/s12959-022-00370-6

**Published:** 2022-03-04

**Authors:** Christy V. John, Rajesh Kumar, Anil Kumar Sivan, Sangeetha Jithin, Rojin Abraham, Chepsy C. Philip

**Affiliations:** Clinical Haematology & Bone Marrow Transplantation, Believers Church Medical College Hospital, Thiruvalla, Kerala 689103 India

## Abstract

**Background:**

Vaccine-induced thrombotic thrombocytopenia (VITT) is a rare but devastating adverse event following adenoviral vector-based vaccinations for COVID-19, resulting in thrombosis, especially of the cerebral and splanchnic vasculature. Despite the progress in laboratory techniques for early diagnosis, VITT remains a clinical diagnosis supplemented by coagulation studies. We report on VITT for the first time from India.

**Case:**

We describe cortical venous sinus thrombosis and intracerebral bleed associated with severe thrombocytopenia in two young men who had no other contributory cause besides a recent ChAdOx1 nCoV-19 vaccination. The diagnosis was supported with PF-4 antibodies in one patient. The second patient’s test could not be processed to technical limitations. Both patients were treated with IVIG at 1 g/kg for 2 days and anticoagulation (Apixaban). One patient fully recovered with no residual deficits, and the other is under treatment and recovering.

**Conclusion:**

VITT can cause devastating fatality and morbidity in otherwise healthy patients via potential immune-mediated effects. Clinicians should have a high suspicion index and treat VITT in the appropriate setting even if the PF-4 antibody testing by ELISA is unavailable or delayed. Though counterintuitive, clinicians must not delay the administration of non-heparin anticoagulation, IVIG and restrict platelet transfusion even in the presence of intracerebral haemorrhage.

**Supplementary Information:**

The online version contains supplementary material available at 10.1186/s12959-022-00370-6.

## Background

Vaccine-induced thrombotic thrombocytopenia (VITT) is a rare but devastating adverse event following the administration of adenovirus-based vaccines, namely the ChAdOx nCoV-19 (Astra Zeneca) and the Ad26.COV2.S (Janssen) [1–3]. The reported cases of VITT from countries with limited resources continue to be scarce despite massive vaccination campaigns. Here we report VITT for the first time from India in two previously healthy young men with prior normal platelet counts who presented with thrombocytopenia, intracerebral bleed, and cerebral venous thrombosis. Delay in diagnosis and instituting appropriate treatment could have been fatal. Both had a favourable outcome on therapy in a limited resource setting without rapid access to confirmatory testing for PF4 antibodies.

## Case 1

A previously healthy 25-year-old male had presented to the emergency department with a history of a subacute onset, progressive headache evolving over the last 6 days. He had been evaluated elsewhere for his symptoms a day earlier. A computed tomography (CT) of the brain revealed a normal study and a hemogram that reported thrombocytopenia with a platelet count of 6 × 10^9^/L. He had no risk factors or suggestive family history for a thrombotic event. He had received the first dose of the ChAdOx1 nCoV-19 (AstraZeneca) vaccine 15 days prior (day of vaccination = day #0 = D0). On the day of presentation (D15), he developed a new-onset weakness of the left half of his body, progressing over the past few hours, evidenced by an inability to sit up from bed and difficulty gripping objects. Neurological examination confirmed a hemiparesis with a hemisensory loss with dysmetria and nystagmus localised to the left.

### Diagnosis

**Fig. 1:**
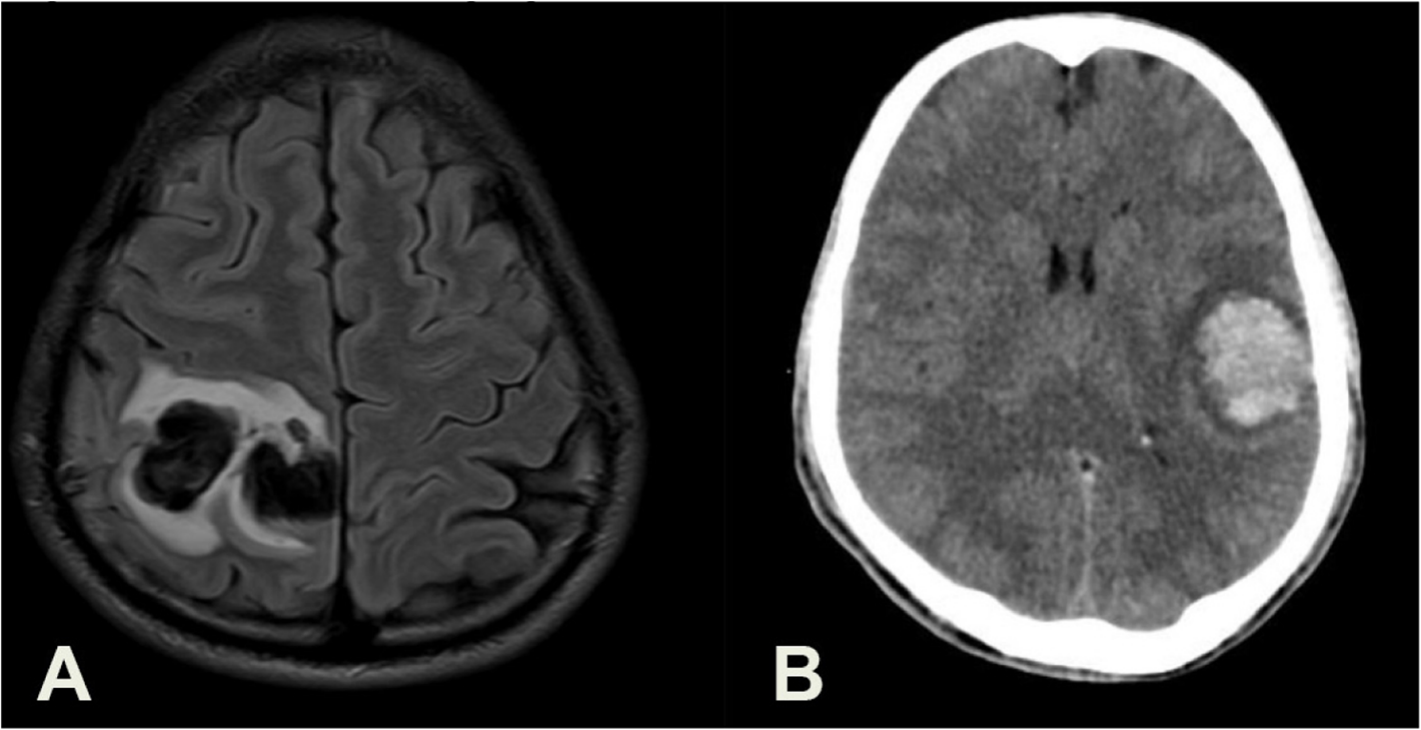
Case 1 Neuroimaging. **A**: Axial FLAIR MR image -right high frontoparietal hematoma (4.7 × 2.3 cm) with oedema. **B**: Plain CT image - worsening with a new hematoma in the left parietal lobe (4.3 × 3.3 cm)

Emergent Magnetic Resonance (MR) Imaging of the brain (Fig. 1) showed a right high parietal haematoma measuring 4.7 × 2.3 cm with oedema and signs of micro haemorrhage in the left parietal lobe and cerebellar hemisphere. Computed tomographic (CT) angiography of the brain revealed extensive thrombosis of the superior sagittal and right transverse sinuses. D-Dimer (Supplementary Figure 1) was elevated (6060 ng/ml) with normal Prothrombin (PT) and activated partial thromboplastin (APTT) time. Testing for antibodies to platelet factor 4 (PF4) was done using a chemiluminescence immunoassay (CLIA) which was negative. Due to the high index of suspicion, it was followed up with Enzyme-Linked Immunosorbent Assay (ELISA) for confirmation of PF4 antibodies. The sample degenerated in transit and was not reported (Test was couriered offsite with a turnaround time of 4 weeks).

### Management

He was managed conservatively. Given the intracerebral haemorrhage (ICH); he received platelet transfusion on admission. With a high index of suspicion, he was initiated on high dose Intravenous Dexamethasone 40 mg (HDD) with Intravenous Immunoglobulin (IVIG) at a dose of 1 g/kg body weight over 24 h. A similar dose of IVIG was administered the following day. He was initiated on anticoagulation with Apixaban 2.5 mg twice daily (in view of thrombocytopenia and the perceived increased risk of bleeding). Despite initial neurological deterioration with motor aphasia and left facial palsy, the dose was incremented to 5 mg twice daily over the next 48 h.

### Follow-up

Over a week, he demonstrated significant clinical improvement corroborated by a brain CT, which showed no signs of haematoma expansion. He demonstrated near-complete resolution of neurological deficits and was self-ambulatory on discharge with the total dissolution of the thrombosis on repeat CT angiography. He remains well on Apixaban with a normal hemogram on follow-up.

## Case 2

A 19-year-old male with no significant medical history was brought to the emergency department after being found in an unresponsive state in his bedroom (D8). His parents recalled his headache for the past 4 days (D5-D8), for which he was evaluated elsewhere with a CT of the brain, which was normal and was given symptomatic care. He had no risk factors or suggestive family history for a thrombotic event. He had received his first dose of ChAdOx1 nCoV-19 (AstraZeneca) 8 days before the onset of symptoms (D0).

### Diagnosis

**Fig. 2:**
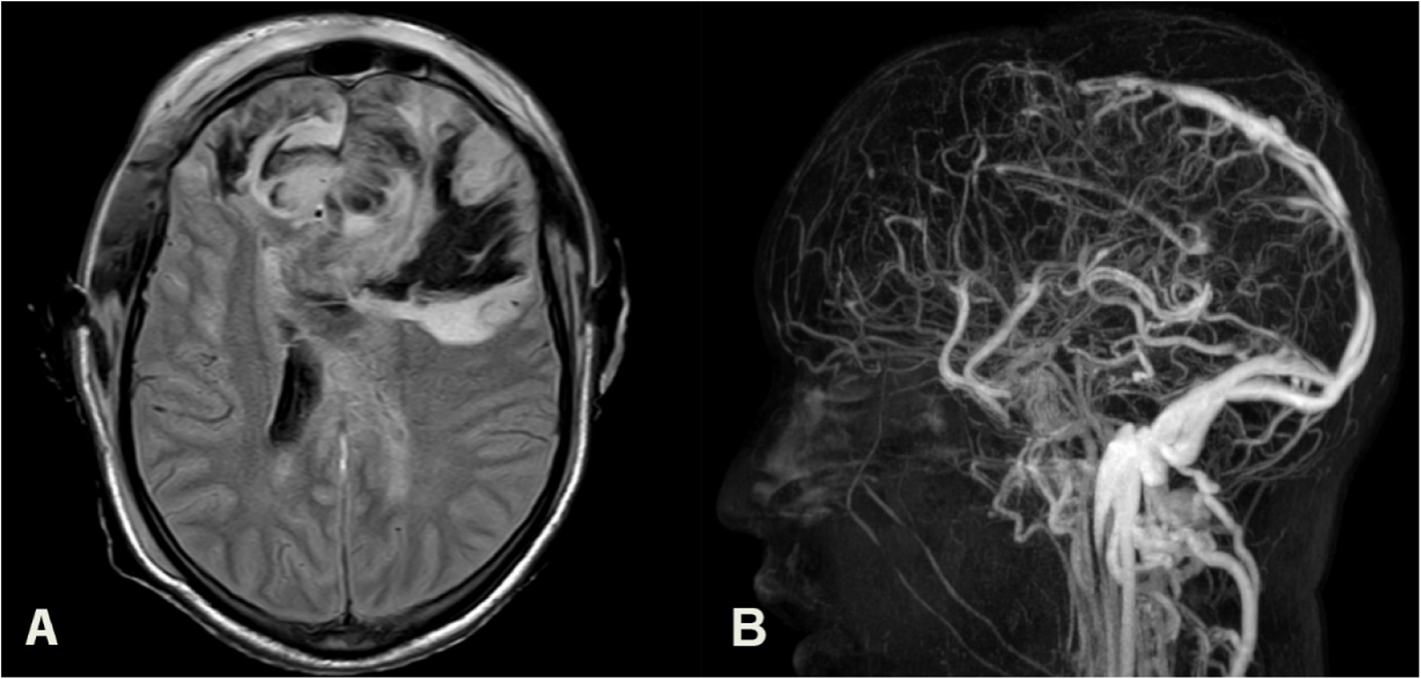
Case 2 Neuroimaging. **A**: Axial MR image -bilateral frontal lobe extensive haemorrhage with intraventricular extension. **B**: MR Venogram showing filling defects in the anterior half of the superior sagittal sinus

Emergent CT imaging of the brain revealed gross hematoma in the bifrontal region. MR Venogram (Fig. 2) confirmed a superior sagittal sinus thrombosis. Laboratory investigations (Supplementary Figure 1) showed thrombocytopenia (4.2 × 10^9/L) and elevated D-Dimer (7760 ng/ml). PT & APTT were normal. Initial testing for anti-PF4 antibody by CLIA was negative, and blood samples sent for confirmatory testing by ELISA were reported positive after 2 months (Supplementary Fig. 2).

### Management

He underwent a life-saving decompressive craniotomy on admission under cover of platelet transfusion with continued post-operative care. Proceeding with a high clinical suspicion of VITT, he was initiated on HDD with IVIG at a dose of 1 g/kg body weight within 48 h of hospitalisation, with a repeat dosing a day later. He was anticoagulated with Apixaban 5 mg twice daily. Over the ensuing 3 days, there was an increment in platelet counts to normal levels without any worsening of any neurological deficits.

### Follow-up

His recovery was complicated by ventilator-associated pneumonia (VAP), bilateral pneumothorax and an extended weaning period with multiple healthcare-associated infections (HAIs) requiring cessation of anticoagulation. However, he is recovering and on follow-up post cranioplasty and is currently on an aggressive physical rehabilitation regimen.

## Discussion

VITT is a rare but devastating adverse event following adenoviral vector-based vaccinations for COVID-19. The most commonly reported symptoms include persisting sudden onset headache, which may be associated with bleeding manifestations and could progress to gross neurological deficits, with an altered mental state [4, 5]. Though majorly reported in unusual anatomic locations (cerebral and splanchnic veins), thrombosis could also involve deep veins, pulmonary embolism and acute arterial thrombosis [6–8]. VITT’s estimated risk is at least 1:100,000 among patients 50 years of age or older and at least 1:50,000 among patients in the younger group [9]. The risk is amplified in younger individuals and recipients of the first dose.

Proposed pathogenetic mechanisms describe a process similar to the autoimmune Heparin Immune Thrombocytopenia (HIT) implicating tetramers of PF4 that crosslink with vaccine proteins to form multimolecular aggregates [10]. The increasing reports of PF4/heparin antibodies in contrast to patients with cerebral venous sinus thrombosis before the COVID-19 pandemic support this mechanism [11]. However, the mere presence of PF4 antibodies need not necessarily imply thrombosis in either HIT or VITT [12, 13]. The presence of EDTA in the vaccine possibly contributes to capillary leakage and dissemination of components in blood. These aggregates are recognised by the Immunoglobulin (Ig) G antibodies and the complement system leading to clustering of PF4 with resulting platelet activation. Cumulative reactions lead to the formation of neutrophil extracellular traps (NETs) with a pan cellular FcγIIa receptor activation akin to HIT. This culminates in a massive coagulation system activation, leading to consumptive coagulopathy with significantly elevated D Dimer levels and hypofibrinogenemia [14]. In contrast to HIT, procoagulant platelets are inhibited by low heparin concentrations and augmented by the addition of PF4 in VITT [15].

Current reports and case definitions highlight the importance of laboratory evidence for VITT. The growing understanding of VITT has improved the assays’ sensitivity, especially the novel PF4 induced platelet activation test (PIPA) being the gold standard in ambiguous cases with low positive or negative PF4 ELISA testing [15, 16]. Although highly sensitive for HIT, rapid testing using CLIA, particle gel immunoassay (PaGIA), and lateral flow assay (LFA) is uniformly inadequate in the diagnosis of VITT [17, 18]. Heparin-induced multi-electrode aggregometry (HIMEA) is a rapid and straightforward alternative to functional assays, which may potentially be helpful in VITT [12]. The combination of a negative CLIA and a positive ELISA, as in our experience, is similar to reports where the administration of IVIG has not impacted the test, likely reflecting that IVIG does not inhibit the VITT binding to PF4 [8, 15].

India has delivered more than 300 million doses to those between 18 and 44 years of age, and the lack of reports on VITT is intriguing. The true incidence of VITT in developing countries is likely under-represented, possibly to stringent case definitions and inadequate laboratory testing facilities. Though the unprecedented rapidity of vaccine production has been integral to the timely response to COVID -19 [19], it is vital that the lack of universally available tests shouldn’t limit surveillance. Therefore, we believe that the interim case definition proposed by the Brighton Collaboration aids in a clinical diagnosis in limited-resource settings despite inaccessibility to adequate laboratory services [20]. The cases we described could be categorised as probable VITT and definite VITT, respectively, as per the NICE guidelines and Level 1 TTS as the Brighton collaboration definitions.

Although current case definitions mandate PF4 testing by ELISA, similar to the recommendations in HIT, timely imaging and treatment must be instituted based on a clinical diagnosis and an individualised basis [21, 22].

### Strength & Limitations

The National Adverse Events Following Immunization (AEFI) Committee in India, which submits data to the Government, reported a total of 179 serious adverse events [no VITT; 3 intracranial bleeds] after the 1st of May [23]. This could be to underreporting of VITT due to lack of such specialised tests or a regional paradox. We recognise the limitations of case reports to generalize and to establish cause-effect relationship; but we report the first confirmed case of VITT from India and this could be of importance to public health and policymakers for this newly recognised entity.

## Conclusion

This is the first report to the best of our knowledge on Vaccine Induced Thrombotic Thrombocytopenia from India with the largest vaccination drive in the world [24]. In our initial and limited experience, VITT typically presents in younger male patients with no prior comorbidities making the severity of illness alarming. While testing for PF4 antibodies adds more evidence to establish the diagnosis, clinicians, though counterintuitive, should not delay the institution of anticoagulation and IVIG while limiting platelet transfusions.

## Supplementary Information

**Additional file 1.**

**Additional file 2.**

## Data Availability

Two supplementary figures attached as supplementary files.

## Abbreviations

AEFI: Adverse Events Following Immunization
ChAdOx: Chimpanzee (Ch) adenovirus-vectored vaccine (Ad), developed at the University of Oxford (Ox)
*CLIA*: Chemiluminescence immunoassay (CLIA)
CT: Computed Tomography
EDTA: Ethylenediaminetetraacetic acid
ELISA: Enzyme-Linked Immunosorbent Assay
FcγIIa: Low-affinity receptor for the constant fragment (Fc) of immunoglobulin (Ig) G
FLAIR: Fluid-attenuated inversion recovery
HAIs: Healthcare-associated infections
HIT: Heparin-induced thrombocytopenia
HIMEA: Heparin-induced multi-electrode aggregometry
ICU: Intensive care unit
IV: Intravenous
IVIG: Intravenous Immunoglobulin
LFA: Lateral flow assay
*MR*: *Magnetic Resonance*
nCoV-19: Novel *coronavirus 19*
NETs: Neutrophil extracellular traps
NICE: National Institute for Health and Care Excellence
PaGIA: Particle gel immunoassay
PF-4: Platelet Factor 4
PIPA: PF4 induced platelet activation test
PT: Prothrombin time
APTT: Activated partial thromboplastin time
VAP: Ventilator-associated pneumonia
VITT: Vaccine-induced Thrombotic Thrombocytopenia
