# Supplementary figures and images for: Vaccine-induced thrombotic thrombocytopenia (VITT): first report from India
Source: Thromb J. 2022 Mar 4;20:11. doi: 10.1186/s12959-022-00370-6 (PMC8894558; doi:10.1186/s12959-022-00370-6)

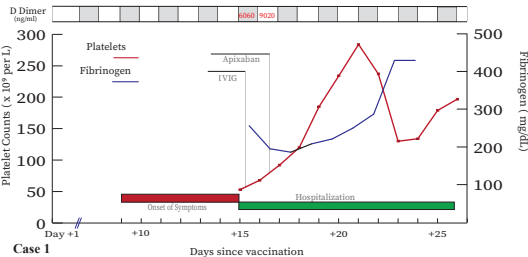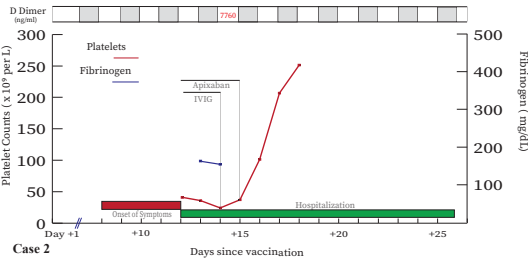

Supplement: Supplementary file 1 — Additional file 1. [file 12959_2022_370_MOESM1_ESM.pdf]

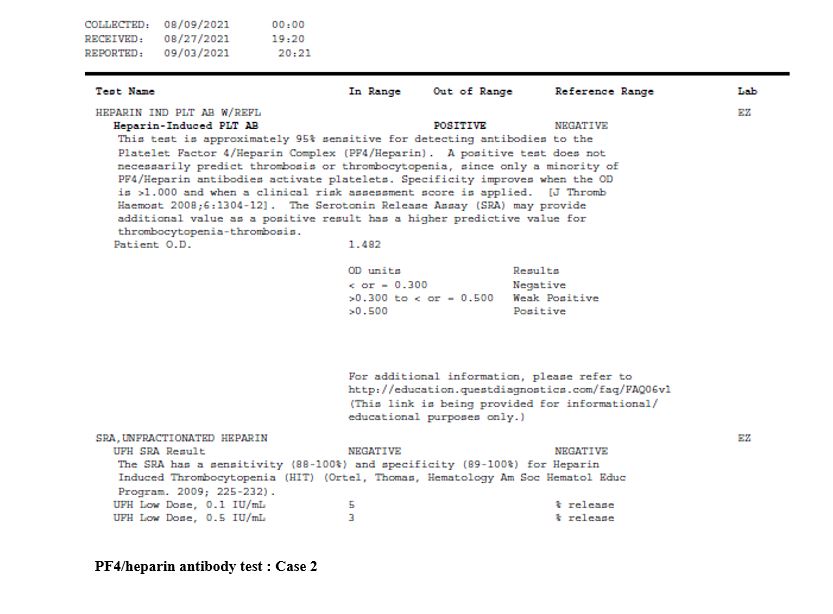

Supplement: Supplementary file 2 — Additional file 2. [file 12959_2022_370_MOESM2_ESM.jpg]
